# Intensity-Dependence of Auditory Evoked Potentials (IDAP) as a Neurophysiological Parameter to Predict Anti-Aggressive Responsiveness to SSRI Treatment

**DOI:** 10.3389/fphar.2021.716338

**Published:** 2021-08-12

**Authors:** Massimiliano Toscano, Alessandro Viganò, Tommaso B Jannini, Marco Ruggiero, Angela Comanducci, Vittorio Di Piero

**Affiliations:** ^1^ Department of Human Neurosciences, “Sapienza” University of Rome, Rome, Italy; ^2^ Department of Neurology, Fatebenefratelli Hospital, Isola Tiberina, Rome, Italy; ^3^ IRCCS Fondazione Don Carlo Gnocchi, Milan, Italy; ^4^ Department of Systems Medicine, University of Rome Tor Vergata, Rome, Italy; ^5^ Physical Medicine and Rehabilitation Division, Umberto I Hospital, Rome, Italy

**Keywords:** impulsive-aggressive behavior, anger, SSRI, intensity-dependence of auditory evoked potentials (IDAP), loudness dependence of auditory evoked potentials (LDAEP)

## Introduction

The rationale for selective serotonin reuptake inhibitor (SSRI) treatment in patients with aggressive behavior comes from several studies aimed at comprehending the neurobiological basis of anger and aggression and, in particular, at investigating the involvement of the serotonergic system in the modulation of offensive aggressive behavior.

The serotonergic system has been originally described as playing an inhibitory control on aggressive impulse. This contributed to define the so-called “serotonergic hypothesis of aggression”, which considers the central serotonergic tone as inversely related to aggression. Reduced concentration levels of both serotonin (5-HT) and its main metabolite, 5-hydoxyindoleacetic acid (5-HIAA), were described in aggressive subjects (Giacalone et al., 1968) as well as in individuals with a history of aggression (Brown et al., 1979) and impulsive aggression (Linnoila et al., 1983).

Understanding the neurobiological aspects of the serotonergic system, and particularly the way how it modulates offensive-aggressive behavior could be of particular relevance from different points of view. First of all, anger, together with aggression (considered its behavioral aspect), is not a mere psychiatric condition, being typically described within the behavioral components of several neurological conditions (Finkel et al., 1996; Kim et al., 2002). Furthermore, aside from mood and behavior, serotonin also modulates a broad spectrum of functions, including cognition, learning, memory, reward processing, sleep, as well as brain development and aging so that, in the last decades, several clinical and brain imaging studies investigated the pathological modifications of serotonergic transmission in Alzheimer Disease and other dementia (Rodríguez et al., 2012; Pourhamzeh et al., 2021). Finally, through its dedicated pathways and interaction with cholinergic, glutamatergic, GABAergic, and dopaminergic transmission systems, abnormal 5-HT neurotransmission has been also described in the early stage of stroke (Rocco et al., 2007) and Parkinson Disease (Huot et al., 2011; Pagano and Politis, 2018), as well as in migraine (Deen et al., 2017) and multiple aspects of epilepsy, including seizure susceptibility, sudden unexpected death, impaired breathing, cardiac function, and arousal during and after seizures (Zhan et al., 2016; Gilliam et al., 2021).

Pharmacological and neurochemical studies implicated serotonergic processes in the regulation of impulse-control disorder (ICD) and related disorders, like aggressive and antisocial personality disorder (Coccaro, 1989), kleptomania, pyromania, intermittent explosive disorder (IED), and pathological gambling (Jannini et al., 2021 May 17. doi: 10.2174/1570159X19666210517150418. Epub ahead of print. PMID: 33998993). Moreover, 5-HT plays a key role as a modulator of impulsive behaviors on the prefrontal cortex, an important hub for cognitive behavior and therefore for the regulation of aggressiveness (New et al., 2004).

Anyway, to date, only a few studies have been conducted to investigate the anti-aggressive effects of SSRI treatment, with fluoxetine 20–60 mg/day and citalopram 20–60 mg/day as the most adopted treatment protocol (Coccaro et al., 1997; Coccaro and Kavoussi, 1997; Coccaro et al., 2009).

A recent meta-analysis on off-label SSRI treatments reports that the evidence on anti-aggressive effects of SSRI treatment, while encouraging, is still poor so far, with weak and even conflicting results (Jannini et al., 2021 May 17. doi: 10.2174/1570159X19666210517150418. Epub ahead of print. PMID: 33998993). In fact, several studies also reported increased aggressive behavior after SSRI treatment (Moore et al., 2010; Park and Lee, 2013; Sharma et al., 2016), suggesting that only a subset of patients are responsive to SSRI treatment. Taken together, these data indicate that only a subgroup of aggressive patients benefits from SSRI treatment, and the responsiveness may depend on intrinsic biochemical differences within impulsive aggressive patients.

In this view, a recent translational study (Peeters et al., 2018) searched for neural (e.g., 5-HT_1A_ receptor density) and behavioral (e.g., baseline aggression and anxiety) parameters that might allow predicting the responsiveness to citalopram in rats. In line with the current literature, the Author reported a dual effect on aggressive behavior, as it both increased and decreased aggressiveness. Even if none of the selected parameters predicted the SSRI treatment effect, we strongly agree with the Author on the need to search for other parameters that may predict anti-aggressive responsiveness to SSRI.

## Cortical Evoked Potentials and aggressive behavior: the Intensity Dependence of Auditory Evoked Potential (IDAP)

From a neurophysiological point of view, many authors firstly investigated the inefficient sensory gating system in self-reported impulsive aggression throughout the cortical evoked potentials (Houston and Stanford, 2001).

The most consistent finding in subjects who show aggressive behavior is the so-called evoked-potentials augmenting-reducing; augmenting-reducing (A-R) paradigm is a measure of sensory inhibition mechanism, based on the reliable individual differences in the change in amplitude with different stimulus intensities (Houston and Stanford, 2001). The evoked potentials augmenting (i.e., a decreased serotonergic tone) is closely related to both the disinhibition aspects of sensation-seeking behavior and impulsive aggressive behavior as well (Carrillo-de-la-Peña, 1992), a “neurophysiological milestone” for the evidence of the involvement of the serotonergic pathway in the aggressive behavior based on cortical evoked potentials.

The intensity dependence of auditory evoked potential (IDAP), also known as “loudness dependence of auditory evoked potential (LDAEP), is a neurophysiological parameter whose rationale came directly from the augmenting-reducing (A-R) paradigm.

Briefly, Auditory Evoked Potentials (AEPs) are evoked by 1,000 Hz tones (50 ms total duration, 10 ms rise and fall times) delivered binaurally through earphones at a random repetition rate of 0.53 ± 0.61 Hz at four different intensities (60, 70, 80, and 90 dB) in a pseudo-randomized order. For each intensity level, 90 trials are collected (sampling frequency 4,000 Hz; sweep duration 400 ms, 50 ms before and 350 ms after the auditory stimulus) (Toscano et al., 2014). The EEG is recorded with an electrode at Cz according to the international 10–20 System and referenced to linked mastoids.

The IDAP is calculated as the linear amplitude/stimulus intensity function (ASF) slope, by measuring the peak-to-peak N1±P2 amplitude (i.e., the maximal negative deflections between 60 and 150 ms post-stimulus, and the positive deflections between 120 and 200 ms, respectively) on four blocks of different stimulus intensity (Hegerl and Juckel, 1993; Rocco et al., 2007; Toscano et al., 2014).

Being modulated by cortical serotonergic innervations, IDAP has been proposed as a reliable neurophysiological marker of the central serotonergic function. Namely, a high firing rate of the serotonergic neurons in the dorsal raphe nuclei, which indicates high serotonergic neurotransmission, is associated with a small increase in the evoked cortical response with increasing loudness of the stimuli, or, in other words, with a weak IDAP. A low central serotonergic neurotransmission, on the other hand, is related to a strong IDAP (Hegerl and Juckel, 1993; Juckel et al., 1999).

Although the close relationship between IDAP and central serotonergic tone is still a matter of debate, further evidence came from studies that reported a functional polymorphism in the promoter region of the serotonin transporter gene (5-HTTLPR) as associated with the auditory evoked potentials’ intensity dependence (Strobel et al., 2003). Many studies also investigated the central nervous system (CNS) effects of drugs acting on 5-HT transmission by means of IDAP (von Knorring and Johansson, 1980; Hegerl et al., 1992; Proietti-Cecchini et al., 1997; Gallinat et al., 2000).

Finally, due to the serotonergic neurotransmission’s role in the modulation of the auditory evoked potentials (AEP), IDAP and other AEP parameters have been adopted as useful non-invasive indicators of cognitive (Buonfiglio et al., 2015) and neuroplastic aspects of serotonergic activity in several psychiatric (Norra et al., 2003; Lee et al., 2005) and neurological conditions (Wang et al., 1996; Rocco et al., 2007; Viganò A et al., 2018).

## Discussion

The treatment of aggressive behavior crucially depends on the understanding of the underlying neurobiological mechanisms.

In our opinion, the main reason for the SSRI effect on aggressive behavior to be such heterogeneous is that the so-called “serotonergic hypothesis of aggression,” which considers the central serotonergic tone as inversely related to aggression, is probably outdated. For instance, trait and state aggression are differentially regulated by the central serotonergic system, since normal offensive aggression is positively related to serotonergic neuronal activity, whereas an inverse relationship probably exists between serotonin activity and impulse-like violent aggression (Coccaro, 1989). This was further confirmed by neurobiological studies that showed, on the one hand, a positive correlation between the level of trait-like aggression and basal cerebrospinal fluid (CSF) concentration of 5-HT and 5-HIAA (i.e., serotonin and its main metabolite, respectively) (van der Vegt et al., 2003), on the other a reduced CSF concentration of 5-HIAA in individuals with impulsive aggression (Linnoila et al., 1983).

Interestingly, in a recent study from our group, we similarly observed through the IDAP opposite 5HT features within patients with aggressive behavior when grouped according to the presence of anger trait or a state condition of anger. (Toscano et al., 2014). This was in sharp contrast with the outdated hypothesis that the activity of 5-HT system is inversely related to the aggressive behavior *tout court*.

Thus, the treatment of aggressive behavior probably depends on the different central serotonergic tone in the treated patients, and the IDAP might be of clinical relevance in the comprehension of the modulating role of serotonin in the pathogenesis of aggressive behavior. In this light, IDAP could represent a useful neurophysiological key to understand what is behind the aggressive patients’ different responsiveness to SSRI treatment.

In this regard, the capability of the IDAP to predict the responsiveness to the SSRI treatment has been already demonstrated in psychiatry in patients with major depressive disorder (i.e., unresponsiveness or even manic switch vs. successful treatment) (Lee et al., 2005; Park and Lee, 2013).

We think it could be of great clinical interest to design RCTs aimed both at clarifying the role of 5HT in aggressive behavior, and at investigating the IDAP usefulness as a neurophysiological marker to predict anti-aggressive responsiveness to SSRI or, in other words, to individuate those aggressive patients who may benefit from the SSRI treatment.
